# The beneficial effect of csDMARDs co-medication on drug persistence of first-line TNF inhibitor in rheumatoid arthritis patients: data from Czech ATTRA registry

**DOI:** 10.1007/s00296-021-05072-2

**Published:** 2022-03-26

**Authors:** Martina Skácelová, Lucie Nekvindová, Heřman Mann, Jakub Závada, Zlatuše Křístková, Jiří Vencovský, Karel Pavelka, Pavel Horák, the ATTRA Registry

**Affiliations:** 1 Third Department of Internal Medicine-Nephrology, Rheumatology and Endrocrinology, Olomouc University Hospital, I.P.Pavlova 6, 77900 Olomouc, Czech Republic; 2 Faculty of Medicine and Dentistry, Palacký University Olomouc, I.P.Pavlova 6, 77900 Olomouc, Czech Republic; 3 Department of Rheumatology, 1st Faculty of Medicine, Charles University, Prague, Czech Republic; 4 Institute of Biostatistics and Analyses, Ltd., Brno, Czech Republic

**Keywords:** Rheumatoid arthritis, TNF inhibitor, csDMARDs, Methotrexate, Registry, Drug persistence

## Abstract

The study aimed to compare treatment retention for first-line TNF inhibitor (TNFi) in the ATTRA registry patients receiving either combination with conventional synthetic DMARDs or TNFi as monotherapy. A retrospective multicenter study analyzed data of all adult patients with rheumatoid arthritis (*n* = 3032) starting TNF inhibitor as the first-line biological therapy in combination with csDMARDs or in monotherapy from January 1st 2012 to December 31st 2020. Kaplan–Meier method was employed to calculate drug retentions. Survival curves of treatment retentions were compared through Log-rank test between the studied subgroups. The hazard ratio for drug discontinuation was assessed through univariate cox regression models. In patients who started the first line TNFi therapy, the median treatment retention was 47.7 (42.2; 53.1) months for combination therapy and 22.7 (14.9; 30.6) months for TNFi monotherapy (*p* < 0.001). Estimated one-year survival was higher in patients on TNFi combined with csDMARDs as compared with TNFi monotherapy (75.3% vs 65.7%); two-year survival rate was 63.2% vs 49.2%, three-year survival rate was 55.4% vs 42.4% and five-year survival 44.9% vs 26.4% of patients. The estimated survival on the first TNFi was higher in patients taking combination therapy with methotrexate than with other csDMARDs (*p* = 0.003). Use of csDMARDs co-medication was associated with significantly better first TNFi drug survival compared to monotherapy. The combination of TNFi with MTX is more effective than the combination with leflunomide, which did not demonstrate a significant effect.

**Supplementary Information:**

The online version contains supplementary material available at 10.1007/s00296-021-05072-2.

## Introduction:

Convential synthetic disease-modifying drugs (csDMARDs) are according to current guidelines first-line drugs for the treatment of rheumatoid arthritis patients. Methotrexate (MTX) is the most common csDMARD used to treat rheumatoid arthritis. According to European League Against Rheumatism (EULAR) recommendations for the management of rheumatoid arthritis (RA), it is the drug of choice that should be administered to all RA patients for whom it is not contraindicated. Where MTX cannot be given, rheumatoid arthritis therapy should be initiated with another csDMARD, most frequently sulfasalazine or leflunomide [1]. Clinical studies have shown that long-term MTX therapy leads to a considerable reduction in swollen and painful joints, significantly decreased inflammatory parameters and generally better outcomes as evaluated by both patients and physicians than treatment with leflunomide [2], gold salts [3] or azathioprine [4]. MTX is associated with the longest treatment retention of all currently used synthetic DMARDs [5], with the most common reasons for drug discontinuation being inadequate effectiveness and toxicity. MTX is used both alone and in combination with glucocorticoids or other csDMARDs; if first-line treatment fails, it should be combined with biological DMARDs (bDMARDs) or targeted synthetic DMARDs (tsDMARDs) [1, 6].

MTX combined with biological agents is more effective than either drug alone; in addition to the synergistic effect, the administration of MTX reduces their immunogenicity [7–12]. While infliximab should be combined with MTX and in the case of golimumab it is strongly recommended as well, other tumor necrosis factor alpha (TNFi) inhibitors may be given in monotherapy. Data both from clinical studies and national registries have shown longer treatment retention for biological agents when combined with MTX than when used alone [13–19].

Despite current guidelines, approximately a third of patients currently taking a bDMARD receives it as monotherapy [20]. There could be a number of contributing objective and subjective factors as intolerance, toxicity or contraindication of csDMARDs, low adherence of patients, shared decision with rheumatologist, etc.

The presented study aimed to compare the real-world data on treatment retention in ATTRA (Anti-TNF Treatment in Rheumatoid Arthritis) registry patients receiving first-line treatment with a TNFi combined with csDMARDs and in those treated with a TNFi alone.

## Methods

A retrospective analysis included all ATTRA (Anti-TNF Treatment in Rheumatoid Arthritis) registry patients with RA (*n* = 3032, female *n* = 2073; 77.3%) started on a first-line TNFi between January 1st 2012 and December 31st 2020. The ATTRA project is a clinical registry under the surveillance of the Czech Society for Rheumatology. It is a multicenter system for assessing the progress and outcomes of biological therapy of inflammatory rheumatic diseases. The observational, depersonalized and anonymous data were collected and stored after obtaining signed written informed consent for data collection from all participants. The registry is approved by the ethical committees (Czech Multicentre Research Ethics Committee, no. 201611 S300, and Institutional Ethics Committee of Institute of Rheumatology, Prague, Czech Republic, no. 10113/2016). The study was performed in accordance with the Helsinki Declaration of 1964, and its later amendments.

The primary endpoints of the study aimed to assess the influence of csDMARDs and namely MTX on the median of TNFi treatment retention and its retention after one, two, three and five years from treatment initiation. The secondary endpoints included assessment of the retention of individual TNFi and comparison of the median of TNFi retention between MTX with other csDMARDS comedication and direct comparison of the effect of MTX and leflunomide.

## Statistical analysis methods

Summary statistics of the mean ± standard deviation (SD) and median with 5th and 95th percentiles were presented for continuous variables. Categorical variables were described through absolute and relative frequencies (i.e. percentages). Statistical comparison of baseline characteristics between the subgroups was made using the non-parametric Mann–Whitney test or non-parametric Kruskal–Wallis test when comparing three groups. After testing of baseline differences through Kruskal–Wallis test, paired comparisons using the Mann–Whitney tests accompanied by Bonferonni correction were applied to discover which two subgroups differ. The relationship between categorical variables was evaluated by the Pearson’s chi-squared test or Fisher exact test when needed.

Retention rates of the first-line treatment were assessed by the Kaplan–Meier method, and the differences in survival curves were examined by the log-rank test. Univariate Cox regression models were employed to quantify the hazard ratio of treatment discontinuation. Bonferroni correction for multiple comparisons was applied when needed. *P* values < 0.05 were considered significant. Analysis was performed using IBM SPSS Statistics 25.0 and R (version 3.5.3).

## Results

### Survival on combination therapy vs monotherapy

Out of the total number of 3032 included, 2682 patients (88.5%) used a TNFi combined with any csDMARD (azathioprine, cyclosporine, hydroxychloroquine, chloroquine, leflunomide, MTX or sulfasalazine), 350 patient used TNFi as monotherapy (11.5%). In the group of patients on MTX the initial mean dose (± SD) was 16.3 ± 5.6 mg, median dose (5th; 95th perc) 15 mg (7.5 mg; 25 mg) weekly. Patients on combination therapy were slightly younger (mean age 53.5 ± 12.9 vs 55.0 ± 13.2; p = 0.036); the mean disease duration was the same (8.1 ± 7.5 years vs 8.4 ± 7.7 years; *p* = 0.962). Combo-group used adalimumab in 43.7%, etanercept in 25.5%, golimumab in 11.4%, certolizumab in 9.9% and infliximab in 9.5%. Bio-originals represented 70.4% of TNFi. Mono-group used adalimumab in 46.9%, etanercept in 31.7%, certolizumab in 9.4%, golimumab in 6.9% and infliximab in 5.1% and bio-originals represented 71.7% of TNFi.

Glucocorticoids were taken by a sizable proportion of patients, particularly those on combination therapy (68.5%), as compared to 59.4% of patients receiving a biological agent alone (*p* < 0.001). The proportions of patients treated with any csDMARDs prior to initiation of first-line biological therapy were also statistically significant, namely 99.1% of combination therapy patients and 92.0% of those receiving monotherapy (*p* < 0.001). There were no major differences in either the numbers of swollen and painful joints or inflammatory parameters between the two groups. Similarly, the Disease Activity Score-28 for RA (DAS-28) with Erythrocyte Sedimentation Rate and the Simple Disease Activity Index at the initiation of TNFi therapy were comparable. Finally, there were no major differences in the EuroQol scale scores. In the Health Assessment Questionnaire Disability Index (HAQ-DI), there was a statistically significant difference between both groups (1.5 ± 0.6 vs 1.6 ± 0.6; *p* = 0.018). The characteristics of the patients and differences between csDMARDs and monotherapy subgroups are shown in Table 1.

**Table 1:** Comparison and characteristics of rheumatoid arthritis patients from ATTRA registry starting first-line TNFi in combination with csDMARDs or as a monotherapy between 1 January 2012 and 31 December 2020

Parameter	Descriptive statistic	CsDMARD co-therapy (*n* = 2682)	*n*	Monotherapy (*n* = 350)	*n*	*P* value
Gender—females	*N* (%)	2073 (77.3%)	2682	275 (78.6%)	350	0.590
Age at diagnosis (years)	Mean ± SD	45.4 (13)	2678	46.7 (13)	337	0.069
	Median (5th; 95th perc.)	45.0 (23.0; 66.0)		48.0 (24.0; 67.0)		
Age at initiation of the 1st biological therapy (years)	Mean ± SD	53.5 (12.9)	2682	55.0 (13.2)	350	**0.036**
	Median (5th; 95th perc.)	55.0 (31.0; 73.0)		56.0 (33.0; 74.0)		
BMI	Mean ± SD	26.9 ± 5.5	2585	26.6 ± 5.2	324	0.348
	Median (5th; 95th perc.)	26.0 (19.5; 37.4)		25.7 (19.5; 36.2)		
Disease duration (years) – from initial symptoms	Mean ± SD	9.8 (8.1)	2522	10.1 ± 8.0	350	0.685
	Median (5th; 95th perc.)	7.6 (1.3; 26.3)		7.7 (1.1; 27.5)		
Disease duration (years) – from diagnosis	Mean ± SD	8.1 (7.5)	2678	8.4 (7.7)	337	0.962
	Median (5th; 95th perc.)	5.9 (0.7; 23.1)		5.9 (0.6; 26.1)		
Glucocorticoid history	*N* (%)	2359 (88.1%)	2678	292 (86.6%)	337	0.444
csDMARD history	*N* (%)	2659 (99.2%)	2662	310 (92.0%)	337	** < 0.001**
Glucocorticoid co-therapy	*N* (%)	1836 (68.5%)	2682	208 (59.4%)	337	** < 0.001**
Seropositivity	*N* (%)	1949 (72.7%)	2681	243 (72.1%)	337	0.819
Anti-CCP	*N* (%)	1871 (71.0%)	2634	218 (66.3%)	329	0.074
ESR (mm/h)	Mean ± SD	34.1 (21.3)	2551	35.8 (24.9)	329	0.864
	Median (5th; 95th perc.)	31.5 (7.0; 79.0)		30.0 (6.0; 86.0)		
CRP (mg/L)	Mean ± SD	21.0 (23.5)	2637	21.7 (23.1)	330	0.786
	Median (5th; 95th perc.)	14.1 (1.3; 64.8)		14.0 (1.2; 68.6)		
No. of painful joints	Mean ± SD	13.5 (5.8)	2681	13.5 (6.2)	334	0.978
	Median (5th; 95th perc.)	13.0 (5.0; 24.0)		13.0 (4.0; 24.0)		
No. of swollen joints	Mean ± SD	9.6 (5.1)	2681	9.4 (5.5)	334	0.278
	Median (5th; 95th perc.)	9.0 (2.0; 19.0)		9.0 (1.0; 20.0)		
PtGA (VAS 0–100)	Mean ± SD	68.6 (20.7)	2678	71.9 (16.8)	333	0.052
	Median (5th; 95th perc.)	72.0 (24.0; 92.0)		75.0 (40.0; 95.0)		
DAS-28-ESR	Mean ± SD	6.1 (0.9)	2549	6.1 (1.0)	328	0.789
	Median (5th; 95th perc.)	6.1 (4.6; 7.6)		6.1 (4.5; 7.9)		
SDAI	Mean ± SD	38.2 (11.3)	2590	38.5 (12.0)	322	0.630
	Median (5th; 95th perc.)	37.2 (21.8; 59.3)		37.7 (20.5; 58.8)		
HAQ-DI	Mean ± SD	1.5 (0.6)	2675	1.6 (0.6)	334	**0.018**
	Median (5th; 95th perc.)	1.5 (0.6; 2.5)		1.6 (0.6; 2.6)		
EuroQol	Mean ± SD	0.3 (0.3)	2663	0.3 (0.3)	334	0.819
	Median (5th; 95th perc.)	0.1 (0.0; 0.8)		0.1 (0.0; 0.8)		

Categorical variables are compared between patient groups using Pearson’s chi-squared test or Fisher exact test when needed. Continuous variables are compared with the non-parametric Mann–Whitney test. The level of statistical significance is 5%

*SD* standard deviation, *csDMARDs* conventional syntetic Disease-Modifying Drugs in Rheumatoid Arthritis, *ESR* erythrocyte sedimentation rate, *CRP* C reactive protein, *DAS28-ESR* Disease Activity Score 28 using ESR, *SDAI* Simple Disease Activity Index; HAQ-DI-Health Assessment Questionnaire-Disability Index, *EuroQoL* European Quality of Life Index, *BMI* body mass index

**Fig. 1:**
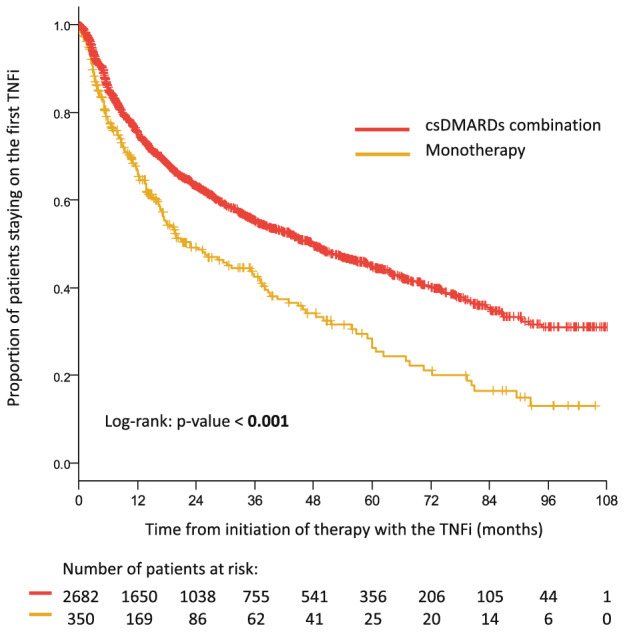
Treatment retention in patients starting the first TNFi in combination with csDMARDs or in monotherapy. The likelihood of treatment retention was estimated using the Kaplan–Meier method. Kaplan–Meier curves for patients treated with TNFi combined with csDMARDs and as a monotherapy were compared using the Log-rank test at the 5% level of a statistical significance

In patients started on first-line therapy with TNFi between January 1st 2012 and December 31st 2020, the estimated median treatment retention was 47.7 (42.2; 53.1) months for combination therapy with csDMARDs and 22.7 (14.8; 30.6) months for TNFi as monotherapy. Estimated one-year survival in patients on TNFi combined with csDMARDs as compared with TNFi monotherapy was 75.3% (95% CI 73.6; 77.1) vs 65.7% (95% CI 60.6; 71.3) of patients; two-year survival rate was 63.2% (95% CI 61.1; 65;2) vs 49.2% (95% CI 43.4; 55.8), three-year survival rate was 55.4% (95% CI 53.2; 57.7) vs 42.4% (95% CI 36.4; 49.5) and five-year survival 44.9% (95% CI 42.5; 47.5) vs 26.4% (95% CI 20.1; 34.6) of patients. Probability of staying on the first TNFi treatment is statistically significantly higher in the group of patients with csDMARDs combination than in patients with monotherapy; Log-rank test: *p* value < 0.001). Patients with csDMARDs combination have around 36% lower risk of TNFi discontinuation than patients with monotherapy (Fig. 1).

During the study, first-line therapy with TNFi in combination with csDMARDs was discontinued by 1127 (42.0%) patients and in monotherapy by 184 (52.6%) patients. The most frequent reason for discontinuation was a loss of drug effect observed in 375 (33.3%) patients on combination therapy and 50 (27.2%) monotherapy patients. Primary ineffectiveness was noted in 220 (19.5%) and 40 (21.7%) patients, respectively. Treatment-related adverse events led to discontinuation in 169 (15.0%) patients receiving combination therapy and 33 (17.9%) monotherapy patients, pharmaco-economic reasons were observed in 90 (8.0%) in combination therapy and in 19 (10.3%) cases in monotherapy. Remission as a reason for discontinuation was noted in 10 patients (0.9%) in the combo group and in 2 (1.1%) in the monotherapy group. Finally, death was noted in 18 (1.6%) cases of combo- and in 2 (1.1%) in monotherapy groups. In 241 (21.4%) combination and in 33 (17.9%) monotherapy patients, the reason for discontinuation was assessed as other or unknown.

### Retention of individual TNFi

The influence of csDMARDs on the retention of the TNFi differed in individual drugs. It was statistically significant in adalimumab and etanercept patients and not in certolizumab, golimumab and infliximab patients. However, these three drugs were rarerly used in monotherapy which might affect the statistical signicicance.

The median of retention of adalimumab in combination with csDMARDs (*n* = 1172) vs adalimumab in monotherapy (*n* = 164) was 58.4 months (95% CI 48.3; 68.5) vs 17.9 months (95% CI 11.4; 24.4). Patients with adalimumab in combination with csDMARDs had a statistically significantly lower risk of treatment discontinuation represented by HR (95% CI) 0.510 (0.406; 0.641) than patients on monotherapy (*p* < 0.001).

The median of retention of etanercept in combination with csDMARDs (*n* = 685) vs etanercept in monotherapy (*n* = 111) was 44.8 months (95% CI 38.4; 51.3) vs 26.3 months (95% CI 8.4; 44.3), which is statistically significant longer for combination therapy with HR (95% CI) 0.699 (0.522; 0.936); *p* = 0.016.

The median of retention of certolizumab in combination with csDMARDs (*n* = 265) vs certolizumab in monotherapy (*n* = 33) was 66.4 months (95% CI 50.7; 82.1) vs 55.8 months (95% CI 18.9; 92.6), which is not significant result for combination therapy (HR (95% CI) 0.774 (0.442; 1.356); *p* = 0.371).

The median of retention of golimumab in combination with csDMARDs (*n* = 306) vs golimumab in monotherapy (*n* = 24) was 47.5 months (95% CI 25.1; 69.8) vs 60.8 months (0,0; 121.7), risk for discontinuation did not differ statistically (HR (95% CI) 0.830 (0.448; 1.540); *p* = 0.555).

The median of retention of infliximab in combination with csDMARDs (*n* = 254) vs infliximab monotherapy (*n* = 18) was 18.9 months (95% CI 14.3; 23.4) vs 15.2 months (95% CI 2.5; 27.8), with no statistical difference (HR (95% CI) 0.796 (0.470; 1.349); *p* = 0.397). Tables summarizing the reasons for the discontinuation of individuals TNFi could be found in the supplementary material (Supplementary Tables 1–5).

### Combination therapy with methotrexate vs other csDMARDs

When taking a closer look at the combination therapy sample comprising 2227 patients on MTX, the median treatment retention was 50.2 (95% CI 43.9; 56.5) months for patients taking TNFi combined with MTX and 35.0 (95% CI 26.1; 44.0) months for individuals on TNFi with other csDMARDs.

**Fig. 2:**
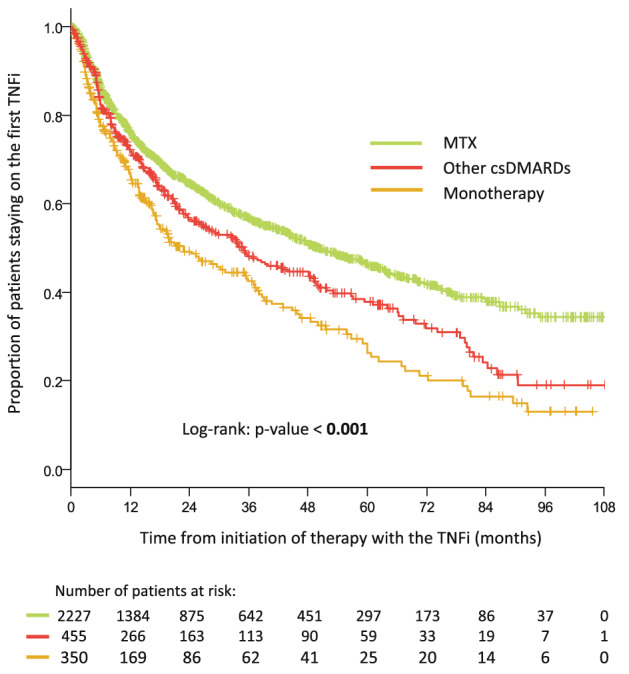
Treatment retention in patients starting the first TNFi in combination with Methotrexate (MTX) other csDMARDs or in monotherapy. The likelihood of treatment retention was estimated using the Kaplan–Meier method. Kaplan–Meier curves for patients treated with TNFi combined with MTX, other csDMARDs and as a monotherapy were compared using the Log-rank test at the 5% level of a statistical significance

The estimated one-year survival rates on therapy accompanied by 95% confidence intervals were 76.0% (74.1; 77.8) for patients taking MTX together with TNFi and 72.3% (68.1; 76.8) for those using other csDMARDs; two-year survival rate was 64.6% (62.4; 66.8) vs 56.4% (51.5; 61.7), three-year survival rate was 56.9% (54.5; 59.4) vs 48.1% (43.0; 53.9) and five-year survival 46.4% (43.7; 49.3) vs 37.8% (32.4; 44.2) of patients. Probability of staying on the first TNFi treatment was statistically significantly higher in both combination groups (MTX, other csDMARDs) than in monotherapy (*p* < 0.001 and *p* = 0.040). Further, MTX combination showed a significantly higher probability of staying on the treatment than other csDMARDs (*p* = 0.003). Patients with MTX co-therapy had 39% lower risk of TNFi discontinuation than patients on monotherapy (*p* < 0.001) and patients with other csDMARDs combination had 21% lower risk of TNFi discontinuation compared to patients on monotherapy (*p* = 0.015). Figure 2 compares retention of first-line TNFi with MTX and with other csDMARDs.

The loss of treatment effect was observed in 287 (31.8%) patients on MTX, 88 (39.1%) patients receiving other csDMARDs. Other reasons for discontinuation represented primary ineffectiveness *n* = 184 (20.4%) vs *n* = 36 (16.0%), adverse events *n* = 139 (15.4%) vs *n* = 30 (13.3%), pharmaco-economic switch *n* = 78 (8.6%) vs *n* = 12 (5.3%), death *n* = 12 (1.3%) vs 6 (2.7%), remission *n* = 7 (0.8%) vs *n* = 3 (1.3%), other or unknown *n* = 192 (21.3%) vs *n* = 49 (21.8%).

### Combination therapy with methotrexate vs leflunomide

**Fig. 3:**
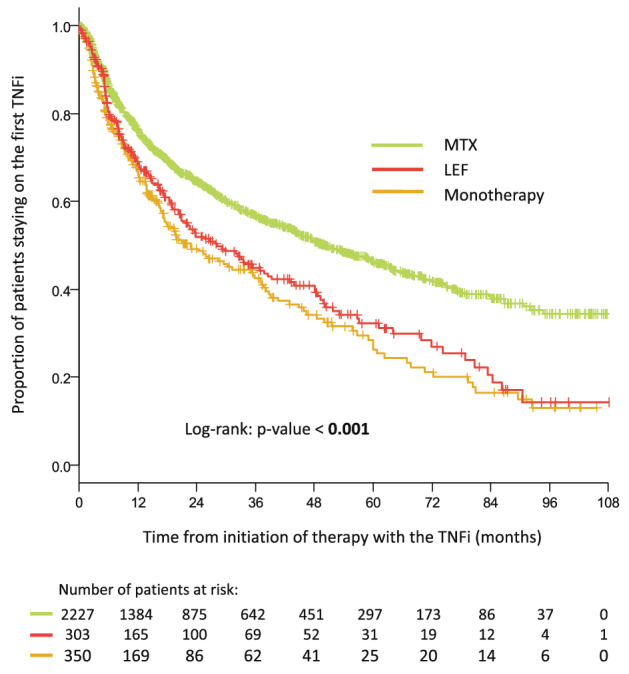
Treatment retention in patients starting the first TNFi in combination with Methotrexate (MTX). Leflunomide (LEF) or in monotherapy. The likelihood of treatment retention was estimated using the Kaplan–Meier method. Kaplan–Meier curves for patients treated with TNFi combined with MTX, LEF and as a monotherapy were compared using the Log-rank test at the 5% level of a statistical significance

The second most frequently used csDMARD was leflunomide (*n* = 303). When comparing treatment retention for first-line TNFi in combination with MTX or LEF, patients receiving TNFi combined with MTX showed higher median retention compared to LEF-50.2 months (95% CI 43.9; 56.5) vs 28.2 months (95% CI 19.3; 37.2), Log-rank: *p* < 0.001. The probability of TNFi retention was slightly higher in LEF group compared to monotherapy, but the difference was not statistically significant (Fig. 3).

Table 2 compares differences in baseline characteristics between patients on TNFi in combination therapy with methotrexate, leflunomide and monotherapy.

**Table 2:** Comparison and characteristics of rheumatoid arthritis patients from ATTRA registry starting first-line TNFi in combination with methotrexate, leflunomide or as a monotherapy between 1 January 2012 and 31 December 2020

Parameter	Descriptive statistic	MTX co-therapy (*n* = 2227)	*n*	LEF co-therapy (*n* = 303)	*n*	Monotherapy (*n* = 350)	*n*	*P* value
Gender—females	*N* (%)	1702 (76.4%)	2227	243 (80.2%)	303	275 (78.6%)	350	0.266
Age at diagnosis (years)	Mean ± SD	45.4 (13.1)	2224	46.2 (11.8)	302	46.7 (13)	337	0.145
	Median (5th; 95th perc.)	46.0 (23.0; 67.0)		46.0 (27.0; 66.0)		48.0 (24.0; 67.0)		
Age at initiation of the 1st biological therapy (years)	Mean ± SD	53.4 (12.9)	2227	55.5 (11.6)	303	55.0 (13.2)	350	**0.009**^**a**^
	Median (5th; 95th perc.)	54.0 (31.0; 73.0)		56.0 (36.0; 74.0)		56.0 (33.0; 74.0)		
BMI	Mean ± SD	27.0 ± 5.5	2144	26.4 ± 5.7	294	26.6 ± 5.2	324	0.076
	Median (5th; 95th perc.)	26.1 (19.5; 37.3)		25.4 (19.1; 38.3)		25.7 (19.5; 36.2)		
Disease duration (years) – from initial symptoms	Mean ± SD	9.7 (8.0)	2093	11.1 (8.3)	283	10.1 ± 8.0	324	**0.005**^**b**^
	Median (5th; 95th perc.)	7.4 (1.3; 26.2)		9.0 (1.7; 28.1)		7.7 (1.1; 27.5)		
Disease duration (years) – from diagnosis	Mean ± SD	8.0 (7.4)	2224	9.3 (7.7)	302	8.4 (7.7)	337	**0.002**^**c**^
	Median (5th; 95th perc.)	5.7 (0.7; 23.0)		7.3 (0.9; 25.0)		5.9 (0.6; 26.1)		
Glucocorticoid history	*N* (%)	1960 (88.1%)	2224	264 (87.4%)	302	292 (86.6%)	337	0.715
csDMARDs history – number								
0	*N* (%)	20 (0.9%)	2210	2 (0.7%)	300	27 (8.0%)	337	< **0.001**†
1	*N* (%)	807 (36.5%)		42 (14.0%)		56 (16.6%)		
2	*N* (%)	644 (29.1%)		94 (31.3%)		101 (30.0%)		
3	*N* (%)	457 (20.7%)		95 (31.7%)		86 (25.5)		
4 or more	*N* (%)	282 (12.8%)		67 (22.3%)		67(19.9)		
Glucocorticoid co-therapy	*N* (%)	1527 (68.6%)	2227	202 (66.7%)	303	208 (59.4%)	350	0.003*
Seropositivity	*N* (%)	1609 (72.2%)	2227	224 (74.2%)	302	243 (72.1%)	337	0.774
Anti-CCP	*N* (%)	1551 (70.9%)	2189	216 (72.5%)	298	218 (66.3%)	329	0.171
ESR (mm/h)	Mean ± SD	34.1 (21.3)	2108	34.7(20.1)	299	35.8 (24.9)	329	0.625
	Median (5th; 95th perc.)	30.0 (7.0; 78.0)		31.0 (6.0;76.0)		30.0 (6.0; 86.0)		
CRP (mg/L)	Mean ± SD	20.9 (23.8)	2183	18.8 (18.2)	302	21.7 (23.1)	330	0.703
	Median (5th; 95th perc.)	14.0 (1.3; 64.4)		14.0 (1.4; 56.0)		14.0 (1.2; 68.6)		
No. of painful joints	Mean ± SD	13.5 (5.8)	2226	13.5 (6.1)	303	13.5 (6.2)	334	0.936
	Median (5th; 95th perc.)	13.0 (5.0; 24.0)		13.0 (5.0; 26.0)		13.0 (4.0; 24.0)		
No. of swollen joints	Mean ± SD	9.7 (5.0)	2226	9.6 (5.3)	303	9.4 (5.5)	334	0.368
	Median (5th; 95th perc.)	9.0 (2.0; 19.0)		9.0 (2.0; 20.0)		9.0 (1.0; 20.0)		
VAS	Mean ± SD	68.4 (21.1)	2223	69.2 (19.3)	303	71.9 (16.8)	333	0.171
	Median (5th; 95th perc.)	72.0 (20.0; 92.0)		70.0 (35.0; 95.0)		75.0 (40.0; 95.0)		
DAS-28-ESR	Mean ± SD	6.1 (0.9)	2106	6.1 (0.9)	299	6.1 (1.0)	328	0.850
	Median (5th; 95th perc.)	6.1 (4.6; 7.6)		6.1 (4.7; 7.7)		6.1 (4.5; 7.9)		
SDAI	Mean ± SD	38.2 (11.2)	2149	38.0 (11.9)	292	38.5 (12.0)	322	0.638
	Median (5th; 95th perc.)	37.4 (21.9; 59.3)		35.4 (21.4; 60.0)		37.7 (20.5; 58.8)		
HAQ-DI	Mean ± SD	1.5 (0.6)	2220	1.6 (0.5)	303	1.6 (0.6)	334	**0.003**^**a**^
	Median (5th; 95th perc.)	1.5 (0.6; 2.4)		1.6 (0.6; 2.5)		1.6 (0.6; 2.6)		
EuroQol	Mean ± SD	0.3 (0.3)	2208	0.2 (0.3)	303	0.3 (0.3)	334	0.357
	Median (5th; 95th perc.)	0.2 (0.0; 0.8)		0.1 (0.0; 0.8)		0.1 (0.0; 0.8)		

Categorical variables are compared between patient groups using Pearson’s chi-squared test or Fisher exact test when needed. Continuous variables are compared with the non-parametric Kruskal–Wallis test. The level of statistical significance is 5%

^a^Paired comparison between the groups using the Mann–Whitney test with the Bonferroni correction: MTX vs LEF co-therapy *p*=0.058; monotherapy vs LEF co-therapy: *p*=1.000; MTX vs monotherapy: *p*=0.073

^b^Paired comparison between the groups using the Mann–Whitney test with the Bonferroni correction: MTX vs LEF co-therapy: *p*=0.004; monotherapy vs LEF co-therapy: *p*=0.163; MTX vs monotherapy: *p*=1.000

^c^Paired comparison between the groups using the Mann–Whitney test with the Bonferroni correction: MTX vs LEF co-therapy: *p*=0.001; monotherapy vs LEF co-therapy: *p*=0.070; MTX vs monotherapy: *p*=1.000

^†^Paired comparison between the groups using Pearson chi-square test with the Bonferroni correction: MTX vs LEF co-therapy: *p*<0.001; monotherapy vs LEF co-therapy: *p*<0.001; MTX vs monotherapy: *p*<0.001

^*^Paired comparison between the groups using Pearson chi-square test with the Bonferroni correction: MTX vs LEF co-therapy: *p*=1.000 monotherapy vs LEF co-therapy: *p*=0.169; MTX vs monotherapy: *p*=0.002

*MTX* methotrexate, *LEF* leflunomide, SD standard deviation, *csDMARDs* conventional syntetic Disease-Modifying Drugs in Rheumatoid Arthritis, *ESR* erythrocyte sedimentation rate, *CRP* C reactive protein, *DAS28-ESR* Disease Activity Score 28 using ESR, *SDAI* Simple Disease Activity Index, *HAQ-DI *Health Assessment Questionnaire-Disability Index, *EuroQoL* European Quality of Life Index, *BMI* body mass index

### Influence of BMI (body mass index) on the TNFi retenciton

The mean and median BMIs for the studied groups are summarized in Tables 1 and 2. Categories of BMI (underweight, normal, overweight, obese) are presented in supplementary materials (Supplementary Tables 6, 7). The effect of BMI category on TNFi retention was not found (Supplementary Fig. 1).

### Drug retention in original versus biosimilar bDMARDs

The bsDMARDs were available for ADA, ETA and INF. Biosimilars were used as first line therapy in 795 (29.6%) patients in the group on combination therapy and in 99 patients (28.3%) in monotherapy. The methotrexate, respectively, leflunomide co-therapy used bsDMARDs in 680 (30.5%), resp. in 80 (26.4%) of cases. The TNFi retention was not statistically different between bo- and bsDMARDs (see Supplementary Fig. 2).

## Discussion

The EULAR recommendations for the management of rheumatoid arthritis recommend the usage of csDMARD as a first-line therapy [1]. Biological DMARD or tsDMARD can be given as a second line if the patient has poor prognostic factors. If the poor prognostic factors are absent changing or adding a csDMARD could be applied. This recommendation for the initialization of bDMARDs or tsDMARDs are widely accepted, nevertheless in different countries are modified to comply with requirements for therapy reimbursement.

The presented retrospective study comes out from the legislation frame for RA therapy coverage in the Czech Republic. The drugs given in the studied period as the first-line biological therapy for RA were TNFi in the vast majority of cases; that is why they were chosen as the matter of interest for this investigation. The treatment was initiated in highly active disease (defined as DAS-28 ≥ 5.1) refractory to at least 6-month therapy with csDMARDs as recommended in the Czech guidelines for RA treatment [21]. Even if current payments rules since 2018 also allowed to cover the therapy cost in patients with moderate active RA (defined as DAS-28 ≥ 3.2), the numbers of these patients in our study are negligible. The purpose of the study was to determine the extent to which the co-medication of csDMARDs with TNFi affects the retention of the first line of biologic therapy, to investigate possible differences between TNFi and to compare MTX with the second most common csDMARD leflunomide.

ATTRA registry do not provide data about the reasons leading to the administration of TNFi in monotherapy, which complicates the interpretation of the results. Even so, the presented data underlined the high importance of co-medication of csDMARDs for better retention of TNFi. The estimated median retention time in patients treated with csDMARDs combination was in this study 47.7 months compared to 22.7 months for monotherapy. Patients with csDMARDs combination had around 36% lower risk of TNFi discontinuation, and their estimated 5-year survival (retention) rate was 45% against only 26% in patients with monotherapy.

The differences that can be deduced from the characteristics of the two sets do not explain these facts. Patients on monotherapy were slightly older, had a slightly higher HAQ, a history of lower exposure to csDMARDs, and lower frequency of co-medication with glucocorticoids. This could indicate their poorer tolerance or compliance with drugs in general, which may then also contribute to worsening of the retention of biologic therapy.

Another interesting finding of this study is the differences between individual TNFi. A significant effect of co-medication on the median retention was seen with adalimumab and etanercept, but not with certolizumab, golimumab or infliximab. This can be explained in the case of golimumab and infliximab used in monotherapy by the error of small numbers, as both drugs should be administered only in combination with csDMARDs as recommended for RA, the monotherapy groups were relatively small (golimumab 24 patients, infliximab 18 patients), which may represent specific situations of this choice.

The positive effect of csDMARDs in this study is driven particularly by MTX, which demonstrated to be more effective in the comparison with other csDMARDs. Surprisingly, the second most commonly used csDMARDs, leflunomide, did not show a statistically significant effect on the median retention of TNF in this study. As data on the reasons for choosing MTX or LEF are not available in our register, we can obtain some insight from the description of both groups. In the case of leflunomide, these RA patients are older, with a longer duration of the disease, with a higher HAQ and with a higher anamnestic number of csDMARDs used before TNFi. This points to severe and more therapeutically resistant forms of the disease, indirectly to MTX intolerance, which appears to negatively affect retention.

There is a lot of data in support of a better effect of TNFi when given in combination with methotrexate, less so with other csDMARDs. A meta-analysis of thirteen randomized trials showed that TNFi combined with MTX was more effective than the biological agents administered as monotherapy [22]. Previously published data from a British Society for Rheumatology Biologics Register (BSRBR) demonstrated that etanercept combined with MTX or another csDMARD was more effective than etanercept alone; in the case of infliximab, however, there was no significant difference in effectiveness between combination therapy with MTX or another csDMARD and monotherapy [23].

Treatment retention analysis seems to be a practical tool for assessing drug effectiveness and safety. Unlike clinical trials, data from registries allow the study of broader patient populations with various drug combinations. In real-world clinical practice, treatment with TNFi tends to be discontinued for various reasons, most frequent adverse effects or a loss of treatment effect [19, 24]. Numerous studies have confirmed that concomitant treatment with MTX or other csDMADRs reduces the risk for discontinuation of biological agents. Data from BSRBR have clearly shown the benefits of combined therapy; in a prospective observational study of 10,396 patients, the median survival of patients taking biological agents was 3.32 years, with treatment retention dropping from 71% in the first year to 42% in the fifth year. Longer treatment retention was observed in patients receiving combination therapy with MTX and at least one other csDMARDs [13], however, this was not confirmed in our study. A long-term Italian study reported 12-year survival in 23.4% of patients taking their first TNFi; concomitant MTX use again was the key factor in the likelihood of longer treatment retention [14]. Out of 2281 participants in the US National Data Bank for Rheumatic Diseases TNFi were discontinued by 1100 (48%) during the first year of their therapy, with MTX being a protective predictor of treatment retention [15]. Similar findings were reported in a Greek observational study [25] and from the Finnish biological therapy registry [26], in which certolizumab and infliximab were most likely to be discontinued. Patients in a Dutch registry receiving combination therapy with TNFi and MTX achieved lower DAS-28, and HAQ-DI scores than their monotherapy counterparts and combination therapy with both MTX and csDMARDs was associated with longer on drug survival [16]. Four-year treatment retention of 42.2% with concomitant MTX use was also demonstrated in GISEA (Group for the Study of Early Arthritis) registry [27]. Over a period of 36 months, 43.9% of patients in ANSWER cohort discontinued treatment with TNFi; the study confirmed the positive effect on retention of combination therapy with MTX [17]. Similarly, Spanish authors reported longer median survival in patients receiving combination therapy with TNFi and MTX as compared with those on combination therapy with other csDMARDs or monotherapy with TNFi alone [18]. MTX as an important factor for treatment retention was confirmed finally by a meta-analysis of drug registries comprising more than 200,000 patients [19].

Evidence for the effect of leflunomide on TNFi retention is not so robust. Data from earlier studies [28–32] show similar effectiveness of LEF and MTX in combination with TNFi, with adverse effect profiles also being similar. A study based on a Swiss registry investigating the effectiveness of TNFi combined with MTX, leflunomide or other csDMARDs also revealed that combination with leflunomide was the second most common approach used in 21% of patients, a greater proportion than in our study where leflunomide with TNFi was taken by 9.9% patients. The median survival of patients receiving stable combination therapy was surprisingly only 16 months as compared to a longer median survival of 31.5 months achieved in patients on csDMARDs alone, suggesting that when therapy failed, csDMARD therapy was more likely to be adjusted first, before replacing the biological drug. There were no major differences in treatment length and effectiveness between the combinations [32]. Strangfeld et al. [33], by contrast, reported shorter treatment retention in patients treated with TNFi (in particular infliximab) combined with leflunomide compared to those taking combination with MTX, with 61.5% of MTX patients and 67.1% of patients on leflunomide discontinuing their therapy after 36 months. Also later data from BSRBR showed different treatment retention depending on various csDMARDs in combination therapy, with longer survival on the first biological agent being observed in patients receiving combination therapy with MTX. Combination therapy with sulfasalazine or leflunomide was associated with an earlier loss of drug effect and treatment discontinuation [13]. This is consistent with data from the ATTRA registry analyzed in the presented study, with the longest median treatment retention for the first TNFi being noted in patients receiving combination therapy with MTX; the median treatment retention was considerably shorter in the case of combination therapy with leflunomide. To a certain extent, these findings suggest that TNFi combination with leflunomide is more likely to be associated with shorter treatment retention. Moreover, Fluori´s study found lower effectiveness and shorter treatment retention for TNFi combined with leflunomide [34].

Besides the main focus of the presented study it also shows some other aspects of bDMARD therapy in patients with RA. First, it should be noted that the persistence of patients with RA on the first TNFi is relatively good and in combination with csDMARDs reaches 75% after the first year and almost 45% after five years of follow-up. In patients in monotherapy, the annual persistence is 65% and 5-year-old 26%. The median survival was 47.7 months for combo group and 22.7 months for monothepray. This also corresponds to the literature sources, for example, the real-life data from a local registry of 583 RA patients on first-line TNFi demonstrated median survival of 53.5 months and overall twelve-year retention 23.4%. Concomitant MTX alo significantly increased TNFi retention [14].

Second, the number of patients starting TNFi in monotherapy was relatively small and represented only 11.5%. Other registries indicate that bDMARDs alone can be given in 30–50% [35, 36]. Our smaller proportion of monothorapy might reflect the compliance efforts of physicians working within the ATTRA registry; but, on the other hand, we do not have the reliable data to verify the actual pick-up of csDMARDs in pharmacies, not to mention the gap in the verification of patients compliance with their use at home. There is a relatively good idea about the administration of TNFi, as these drugs are registered separately and drug count is performed at the visits. Thus, it could be assumed that the numbers of patients using TNFi in monotherapy are probably higher than reported.

Last, but not least, a relatively large percentage of patients initiates TNFi in combination with glucocorticoids (68.5% in the combo group, 59.4% in monotherapy.) The current recommendations are restrictive in terms of the length of glucocorticoids (GCs) administration. It is evident that chronic GCs therapy is administered relatively frequently in RA, although with a trend to decrease their administration in recent years [37]. In our case, this indicates in particular the high activity of patients at the start of TNFi. Whereas data on discontinuation of GCs therapy during bDMARD therapy are not complete in the ATTRA registry, it cannot be reliably interpreted. We would like to focus on the coming years and to collect also this data. It should also be noted that discussions on the effect and risks of long-term low-dose GCs therapy in RA are far from over. Some patients will still prefer monotherapy with low-dose GCs instead of low-dose MTX, especially in established disease and GCs dependent disease [38]. We expect early results of a randomized, double-blind, clinical trial GLORIA (Glucocorticoid Low-dose Outcome in RA) which evaluates the safety and the effectiveness of low dose GCs (5 mg per day of prednisolone versus placebo) in elderly RA patients over 65 years [39, 40].

There are some limitations of the presented study. This is an analysis of real-world database from the registry, the absence of any randomization is a limiting factor in the interpretation of differences between individual TNFi. No data is available on the reasons that led physicians in individual cases to decide to administer TNFi alone or in combination with csDMARDs and no attempt was made to compare patients with similar input parameters. The intial activity of RA seems to be considerably high. In the years 2012–2018 the bDMARDs were reserved in Chech republic for RA patients with high disease activity despite csDMARDs treatment. Even if we cannot exclude a possible up-scoring in individual cases, we are convinced that this is only a marginal problem which did not influence the results of our observations. Since 2018 the bDMARDs are available in Czech Republic also for moderate RA activity and we think that possible up-scoring will play less and less important role. The trend towards earlier switches could be influenced also by the availability of other drugs with a same or different modes of action. In 2012, all five TNFi plus abatacept, tocilizumab and rituximab were available in the Czech Republic. Later also sarilumab became the choice. The possibilities of the switches were relatively extensive throughout the duration of our data collection. The advent of JAK inhibitors has expanded the range of drugs available in recent years. Sadly, in the Czech drug evaluation system, determining the reimbursement of a drug takes a long time. As a result, JAK inhibitors have only become more widely available since 2019. It can be assumed that expanding the range of drugs will motivate physicians and patients to more frequent switches, which could affect drug retention. However, in our opinion, only the following years will show whether it will be seen in ATTRA register as well.

To conclude, the authors are convinced the study provides strong evidence that patients receiving TNFi in combination with csDMARDs have significantly better retention of first-line biologic therapy. This effect is mainly driven by MTX administration in most cases, but a statistically significant difference is also present in the set of other csDMARDs. Surprisingly, it was not confirmed for leflunomide. Another interesting finding is the difference in the effect of co-medication on the retention of individual TNFi, from which no broader conclusions can be drawn, since this requires the support of other observational data.

## Supplementary Information

Below is the link to the electronic supplementary material.

Supplementary file1 (DOC 45 KB)

Supplementary file2 (DOC 46 KB)

Supplementary file3 (DOC 45 KB)

Supplementary file4 (DOC 44 KB)

Supplementary file5 (DOC 43 KB)

Supplementary file6 (DOC 50 KB)

Supplementary file7 (DOC 52 KB)

Supplementary file8 Supplementary Fig. 1 Treatment retention in patients starting the first TNFi according the BMI (Body mass index) structure. The likelihood of treatment retention was estimated using the Kaplan-Meier method. Kaplan-Meier curves for patients treated with TNFi (Norma/underweight, overweight, obese) were compared using the Log-rank test at the 5% level of a statistical significance (EPS 381 KB)

Supplementary file9 Supplementary Fig. 2 Treatment retention in patients starting the first TNFi – comparison of bo- and bsDMARDs. The likelihood of treatment retention was estimated using the Kaplan-Meier method. Kaplan-Meier curves for patients treated with boDMDARDs vs bsDMARDS were compared using the Log-rank test at the 5% level of a statistical significance (EPS 366 KB)

## Data Availability

The datasets generated and analyzed during the current study are available from the corresponding author on reasonable request.
