# Supplementary material for: The beneficial effect of csDMARDs co-medication on drug persistence of first-line TNF inhibitor in rheumatoid arthritis patients: data from Czech ATTRA registry
Source: Rheumatol Int. 2022 Mar 26;42(5):803–14. doi: 10.1007/s00296-021-05072-2 (PMC9007799; doi:10.1007/s00296-021-05072-2)
Supplement: Supplementary file 1 — Supplementary file1 (DOC 45 KB) [file 296_2021_5072_MOESM1_ESM.doc]

**Supplementary Table 1.**

Reasons for adalimumab discontinuation in (patients starting 1st-line in 2012 or later)

| **Reasons for discontinuation** (n=530) | **MTX in combination** (n=353) | **Other csDMARDs in combination** (n=87) | **Monotherapy** (n=90) |
| --- | --- | --- | --- |
| **Loss of effect** (secondary failure) | 110 (31.2%) | 38 (43.7%) | 22 (24.4%) |
| **Inefficacy** (primary failure) | 78 (22.1%) | 17 (19.5%) | 23 (25.6%) |
| **Adverse event** | 61 (17.3%) | 11 (12.6%) | 15 (16.7%) |
| **Pharmaco-economic reasons** | 29 (8.2%) | 4 (4.6%) | 11 (12.2%) |
| **Remission** | 5 (1.4%) | 0 (0.0%) | 2 (2.2%) |
| **Death** | 3 (0.8%) | 3 (3.4%) | 0 (0.0%) |
| **Patient cannot be contacted** | 3 (0.8%) | 0 (0.0%) | 2 (2.2%) |
| **Other** | 64 (18.1%) | 14 (16.1%) | 15 (16.7%) |
