# Supplementary material for: The beneficial effect of csDMARDs co-medication on drug persistence of first-line TNF inhibitor in rheumatoid arthritis patients: data from Czech ATTRA registry
Source: Rheumatol Int. 2022 Mar 26;42(5):803–14. doi: 10.1007/s00296-021-05072-2 (PMC9007799; doi:10.1007/s00296-021-05072-2)
Supplement: Supplementary file 2 — Supplementary file2 (DOC 46 KB) [file 296_2021_5072_MOESM2_ESM.doc]

**Supplementary Table 2.**

Reasons for etanercept discontinuation in (patients starting 1st-line in 2012 or later)

| **Reasons for discontinuation** (n=333) | **MTX in combination** (n=210) | **Other csDMARDs in combination** (n=69) | **Monotherapy** (n=54) |
| --- | --- | --- | --- |
| **Loss of effect** (secondary failure) | 48 (22.9%) | 22 (31.9%) | 13 (24.1%) |
| **Inefficacy** (primary failure) | 37 (17.6%) | 9 (13.0%) | 11 (20.4%) |
| **Pharmaco-economic reasons** | 33 (15.7%) | 6 (8.7%) | 6 (11.1%) |
| **Adverse events** | 33 (15.7%) | 6 (8.7%) | 9 (16.7%) |
| **Death** | 2 (1.0%) | 2 (2.9%) | 1 (1.9%) |
| **Remission** | 1 (0.5%) | 1 (1.4%) | 0 (0.0%) |
| **Patient cannot be contacted** | 0 (0.0%) | 1 (1.4%) | 2 (3.7%) |
| **Other** | 56 (26.7%) | 22 (31.9%) | 12 (22.2%) |
