# Supplementary material for: The beneficial effect of csDMARDs co-medication on drug persistence of first-line TNF inhibitor in rheumatoid arthritis patients: data from Czech ATTRA registry
Source: Rheumatol Int. 2022 Mar 26;42(5):803–14. doi: 10.1007/s00296-021-05072-2 (PMC9007799; doi:10.1007/s00296-021-05072-2)
Supplement: Supplementary file 3 — Supplementary file3 (DOC 45 KB) [file 296_2021_5072_MOESM3_ESM.doc]

**Supplementary Table 3.**

Reasons for certolizumab discontinuation in (patients starting 1st-line in 2012 or later)

| **Reasons for discontinuation** (n=118) | **MTX in combination** (n=75) | **Other csDMARDs in combination** (n=29) | **Monotherapy** (n=14) |
| --- | --- | --- | --- |
| **Loss of effect** (secondary failure) | 37 (49.3%) | 10 (34.5%) | 5 (35.7%) |
| **Inefficacy** (primary failure) | 13 (17.3%) | 5 (17.2%) | 2 (14.3%) |
| **Adverse event** | 8 (10.7%) | 4 (13.8%) | 3 (21.4%) |
| **Pharmaco-economic reasons** | 3 (4.0%) | 2 (6.9%) | 0 (0.0%) |
| **Death** | 2 (2.7%) | 0 (0.0%) | 1 (7.1%) |
| **Patient cannot be contacted** | 0 (0.0%) | 0 (0.0%) | 1 (7.1%) |
| **Remission** | 0 (0.0%) | 1 (3.4%) | 0 (0.0%) |
| **Other** | 12 (16.0%) | 7 (24.1%) | 2 (14.3%) |
