# Supplementary material for: The beneficial effect of csDMARDs co-medication on drug persistence of first-line TNF inhibitor in rheumatoid arthritis patients: data from Czech ATTRA registry
Source: Rheumatol Int. 2022 Mar 26;42(5):803–14. doi: 10.1007/s00296-021-05072-2 (PMC9007799; doi:10.1007/s00296-021-05072-2)
Supplement: Supplementary file 4 — Supplementary file4 (DOC 44 KB) [file 296_2021_5072_MOESM4_ESM.doc]

**Supplementary Table 4.**

Reasons for golimumab discontinuation in (patients starting 1st-line in 2012 or later)

| **Reasons for discontinuation** (n=136) | **MTX in combination** (n=102) | **Other csDMARDs in combination** (n=23) | **Monotherapy** (n=11) |
| --- | --- | --- | --- |
| **Loss of effect** (secondary failure) | 42 (41.2%) | 10 (43.5%) | 7 (63.6%) |
| **Inefficacy** (primary failure) | 28 (27.5%) | 3 (13.0%) | 4 (36.4%) |
| **Adverse events** | 11 (10.8%) | 4 (17.4%) | 0 (0.0%) |
| **Death** | 3 (2.9%) | 1 (4.3%) | 0 (0.0%) |
| **Pharmaco-economic reasons** | 2 (2.0%) | 0 (0.0%) | 0 (0.0%) |
| **Remission** | 1 (1.0%) | 1 (4.3%) | 0 (0.0%) |
| **Other** | 15 (14.7%) | 4 (17.4%) | 0 (0.0%) |
