# Supplementary material for: The beneficial effect of csDMARDs co-medication on drug persistence of first-line TNF inhibitor in rheumatoid arthritis patients: data from Czech ATTRA registry
Source: Rheumatol Int. 2022 Mar 26;42(5):803–14. doi: 10.1007/s00296-021-05072-2 (PMC9007799; doi:10.1007/s00296-021-05072-2)
Supplement: Supplementary file 5 — Supplementary file5 (DOC 43 KB) [file 296_2021_5072_MOESM5_ESM.doc]

**Supplementary Table 5.**

Reasons for infliximab discontinuation in (patients starting 1st-line in 2012 or later)

| **Reasons for discontinuation** (n=194) | **MTX in combination** (n=162) | **Other csDMARDs in combination** (n=17) | **Monotherapy** (n=15) |
| --- | --- | --- | --- |
| **Loss of effect** (secondary failure) | 50 (30.9%) | 8 (47.1%) | 3 (20.0%) |
| **Inefficacy** (primary failure) | 28 (17.3%) | 2 (11.8%) | 0 (0.0%) |
| **Adverse events** | 26 (16.0%) | 5 (29.4%) | 6 (40.0%) |
| **Pharmaco-economic reasons** | 11 (6.8%) | 0 (0.0%) | 2 (13.3%) |
| **Death** | 2 (1.2%) | 0 (0.0%) | 0 (0.0%) |
| **Other** | 45 (27.8%) | 2 (11.8%) | 4 (26.7%) |
