# Supplementary material for: The beneficial effect of csDMARDs co-medication on drug persistence of first-line TNF inhibitor in rheumatoid arthritis patients: data from Czech ATTRA registry
Source: Rheumatol Int. 2022 Mar 26;42(5):803–14. doi: 10.1007/s00296-021-05072-2 (PMC9007799; doi:10.1007/s00296-021-05072-2)
Supplement: Supplementary file 6 — Supplementary file6 (DOC 50 KB) [file 296_2021_5072_MOESM6_ESM.doc]

**Supplementary Table 6.**

Body mass index and its structure at the baseline in csDMARDs and monotherapy groups

| **Parameter** | **Descriptive statistic** | **csDMARD co-therapy** (n=2682) | **n** | **Monotherapy** (n=350) | **n** | **P-value** |
| --- | --- | --- | --- | --- | --- | --- |
| **BMI** | Mean ± SD | 26.9 ± 5.5 | 2585 | 26.6 ± 5.2 | 324 | 0.348 |
|  | Median (5th; 95th perc.) | 26.0 (19.5; 37.4) |  | 25.7 (19.5; 36.2) |  |  |
| Underweight (<18.5) | n (%) | 70 (2.7 %) | 2585 | 6 (1.9 %) | 324 | 0.846 |
| Normal (18.5–24.9) | n (%) | 1010 (39.1 %) |  | 129 (39.8 %) |  |  |
| Overweight (25–29.9) | n (%) | 844 (32.6 %) |  | 112 (34.6 %) |  |  |
| Obesity class I (30–34.9) | n (%) | 430 (16.6 %) |  | 53 (16.4 %) |  |  |
| Obesity class II (35–39.9) | n (%) | 167 (6.5 %) |  | 18 (5.6 %) |  |  |
| Obesity class III (≥40) | n (%) | 64 (2.5 %) |  | 6 (1.9 %) |  |  |
| Underweight + normal (<25) | n (%) | 1080 (41.8 %) |  | 135 (41.7 %) |  |  |
| Overweight (25–29.9) | n (%) | 844 (32.6 %) | 2585 | 112 (34.6 %) | 324 | 0.707 |
| Obesity (≥30) | n (%) | 661 (25.6 %) |  | 77 (23.8 %) |  |  |
