# Supplementary material for: The beneficial effect of csDMARDs co-medication on drug persistence of first-line TNF inhibitor in rheumatoid arthritis patients: data from Czech ATTRA registry
Source: Rheumatol Int. 2022 Mar 26;42(5):803–14. doi: 10.1007/s00296-021-05072-2 (PMC9007799; doi:10.1007/s00296-021-05072-2)
Supplement: Supplementary file 7 — Supplementary file7 (DOC 52 KB) [file 296_2021_5072_MOESM7_ESM.doc]

**Supplementary Table 7.**

Body mass index and its structure in MTX, LEF and monotherapy group at the baseline

| **Parameter** | **Descriptive statistic** | **MTX co-therapy** (n=2227) | **n** | **LEF co-therapy** (n=303) | **n** | **Monotherapy** (n=350) | **n** | **P-value** |
| --- | --- | --- | --- | --- | --- | --- | --- | --- |
| **BMI** | Mean ± SD | 27.0 ± 5.5 | 2144 | 26.4 ± 5.7 | 294 | 26.6 ± 5.2 | 324 | 0.076 |
|  | Median (5th; 95th perc.) | 26.1 (19.5; 37.3) |  | 25.4 (19.1; 38.3) |  | 25.7 (19.5; 36.2) |  |  |
| Underweight (<18.5) | n (%) | 57 (2.7 %) | 2144 | 10 (3.4 %) | 294 | 6 (1.9 %) | 324 | 0.543 |
| Normal (18.5–24.9) | n (%) | 818 (38.2 %) |  | 127 (43.2 %) |  | 129 (39.8 %) |  |  |
| Overweight (25–29.9) | n (%) | 704 (32.8 %) |  | 95 (32.3 %) |  | 112 (34.6 %) |  |  |
| Obesity class I (30–34.9) | n (%) | 368 (17.2 %) |  | 38 (12.9 %) |  | 53 (16.4 %) |  |  |
| Obesity class II (35–39.9) | n (%) | 146 (6.8 %) |  | 15 (5.1 %) |  | 18 (5.6 %) |  |  |
| Obesity class III (≥40) | n (%) | 51 (2.4 %) |  | 9 (3.1 %) |  | 6 (1.9 %) |  |  |
| Underweight + normal (<25) | n (%) | 875 (40.8 %) |  | 137 (46.6 %) |  | 135 (41.7 %) |  |  |
| Overweight (25–29.9) | n (%) | 704 (32.8 %) | 2144 | 95 (32.3 %) | 294 | 112 (34.6 %) | 324 | 0.229 |
| Obesity (≥30) | n (%) | 565 (26.4 %) |  | 62 (21.1 %) |  | 77 (23.8 %) |  |  |
